# Granulomatous infiltration of a parathyroid adenoma presenting as primary hyperparathyroidism in a woman: a case report

**DOI:** 10.1186/1752-1947-4-400

**Published:** 2010-12-09

**Authors:** İnan Anaforoğlu, Çiğdem Şiviloğlu, Ayten Livaoğlu, Ekrem Algün

**Affiliations:** 1 Department of Endocrinology and Metabolism, Trabzon Numune Training and Research Hospital, Trabzon, 61000, Turkey; 2 Department of Pathology, Trabzon Numune Training and Research Hospital, Trabzon, 61000, Turkey

## Abstract

**Introduction:**

Hypercalcemia can be associated with vitamin D (1,25(OH)_2_D_3_) -mediated granulomatous disorders in addition to primary hyperparathyroidism (PHPT). Although most patients with granulomatous disease-related hypercalcemia are asymptomatic, symptoms and signs of chronic hypercalcemia can occur. There are many reports about co-presentation of a parathyroid adenoma and a granulomatous disorder in the literature. However, granulomatous inflammation within a parathyroid adenoma is very rare.

**Case presentation:**

A 50-year-old Caucasian woman presented with generalized bone pain and muscular weakness. Biochemical findings suggested PHPT. She underwent excision of an enlarged right inferior parathyroid gland. Histopathological analysis revealed features of a parathyroid adenoma with foci of epithelioid non-caseating granulomas. The etiology of the granulomatous infiltration could not be determined. She is still normocalcemic at the ninth month after surgery and is being followed for the possible manifestation of an occult disease.

**Conclusion:**

Granulomatous infiltration of a parathyroid adenoma is a rare condition. Pathological examination of the excised adenoma is the only way to diagnose the underlying occult granulomatous disorder. Clinicians should also consider persistent hypercalcemia to be a possible indicator of concomitant parathyroid adenoma.

## Introduction

Accelerated bone resorption, excessive gastrointestinal absorption of calcium, or decreased renal excretion of calcium can all result in hypercalcemia individually. In some disorders, however, more than one mechanism may be involved. Primary hyperparathyroidism (PHPT) is the most common cause of hypercalcemia among outpatients, whereas malignancy is the leading underlying disorder in hospital cases. Solitary parathyroid adenoma followed by parathyroid hyperplasia and carcinoma are the most frequent causes of PHPT [1].

Hypercalcemia in PHPT reflects parathormone-mediated activation of osteoclasts, leading to increased bone resorption [1]. It has also been demonstrated to be associated with vitamin D (1,25(OH)_2_D_3_) mediated granulomatous disorders, reflecting disordered extrarenal production of 1,25(OH)_2_D_3 _[2]. The most common granulomatous disorders causing hypercalcemia are sarcoidosis and tuberculosis. Other non-infectious causes include lymphoma, silicone-induced granulomatosis, paraffin-induced granulomatosis, berylliosis, Wegener's granulomatosis, eosinophilic granuloma, and infantile fat necrosis. Underlying infectious etiologies may include candidiasis, leprosy, histoplasmosis, coccidioidomycosis, and cat-scratch disease [2]. Although most patients with granulomatous disease-related hypercalcemia are asymptomatic, symptoms and signs of chronic hypercalcemia can occur [2]. Herein we report a patient with PHPT who was detected to have non-caseating granulomas within her parathyroid adenoma.

## Case Presentation

A 50-year-old Caucasian woman presented with generalized bone pain and weakness. She reported a subtotal thyroidectomy operation for her multinodular goiter 20 years before admission. Her medical history included hypertension, for which she was taking amlodipine 10 mg daily, and recurrent nephrolithiasis. Her physical examination was normal, and she seemed to be euthyroid at presentation.

Her biochemical findings were suggestive of PHPT (Table 1). Abdominal ultrasonography demonstrated bilateral pelvicaliectasis and microcalculi of the right kidney. Bone mineral density was osteopenic at her spine and hip. Neck ultrasonography showed heterogeneous thyroid parenchyma and a hypoechoic lesion at the inferior region of her right thyroid lobe compatible with a parathyroid adenoma.

**Table 1:** Laboratory Findings Before and After the Excision of the Parathyroid Adenoma

Variable Level	Before Operation	After Operation	Normal Range
Calcium, mg/dl	11.5-12	10.1-9	8.2-11
Phosphorous, mg/dl	3.1	3.7	2.5-4.5
24-hour urinary calcium, mg/day*	340.8	60	200
Parathormone, pg/ml	105.3-98.5	40.8	15-65
25 OH vitamin D, pg/ml	n.d.	34.7	10-80
1,25(OH)_2_D_3_, pg/ml	51.7	27.1	16-65

All measurements were performed in serum, except the calcium in urine (marked as *). n.d. indicates not detected.

**Figure 1:**
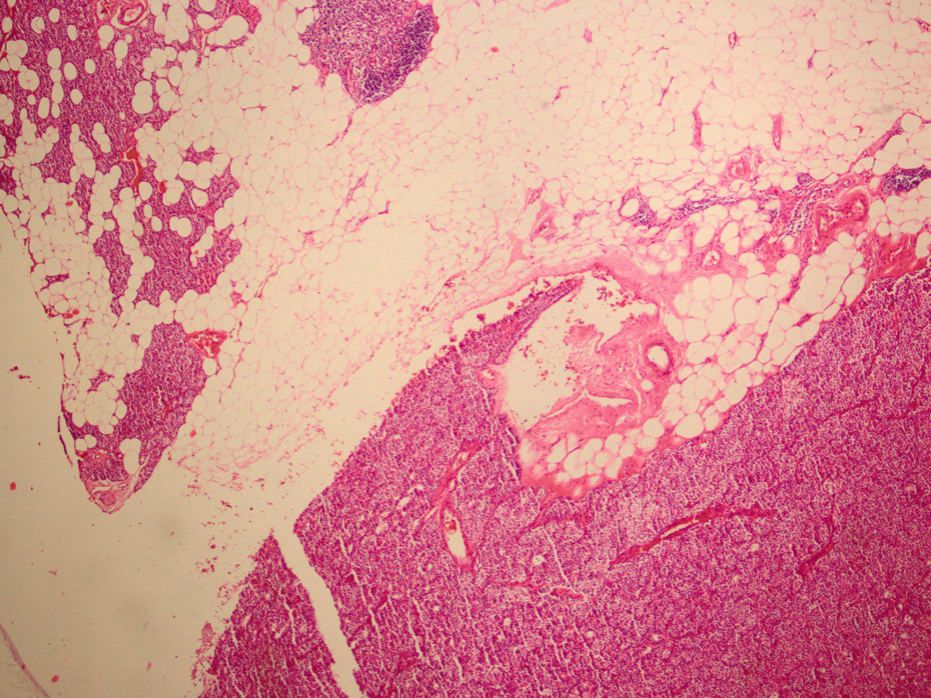
**Photomicrograph of hematoxylin and eosin-stained slide preparation demonstrating the parathyroid gland and the rim of our patient's adenoma**.

**Figure 2:**
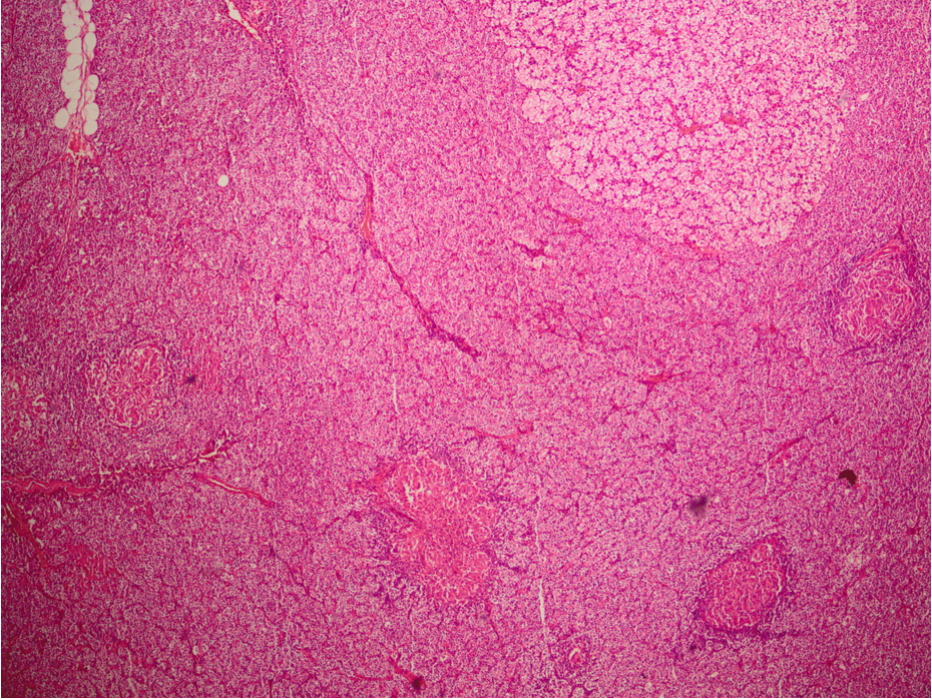
**Photomicrograph exhibiting granulomas within our patient's parathyroid adenoma**.

**Figure 3:**
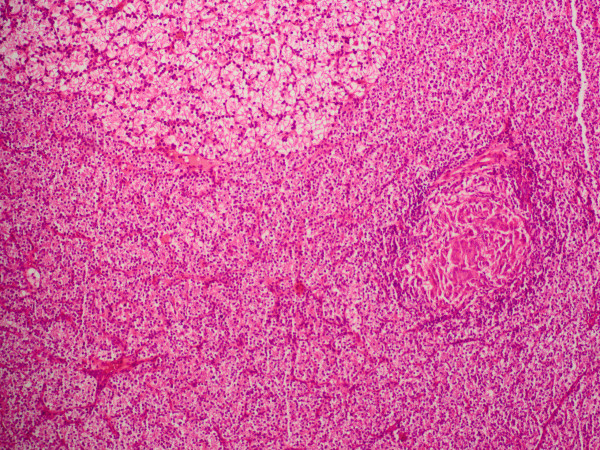
**Photomicrograph exhibiting granulomas within our patient's parathyroid adenoma**.

Under a presumptive diagnosis of PHPT, she underwent excision of the enlarged right inferior parathyroid adenoma. The excised mass was well defined with a yellow-pink cut surface and was measured as 20 mm × 15 mm × 10 mm on gross examination. Histopathological examination revealed a parathyroid adenoma with foci of epithelioid granuloma representing non-caseating granulomas (Figure 1). There were epithelioid histiocytes and multinuclear giant cells tending to come together, with lenfoid cells surrounding the granulomas (Figures 2 and 3). Two of her lymph nodes were compatible with reactive hyperplasia.

Possible underlying pathophysiological disorders for non-caseating granuloma formation were sought thoroughly. She was completely asymptomatic for tuberculosis, and a polymerase chain reaction (PCR) assay for *Mycobacterium tuberculosis *of the homogenates of the parathyroid tumor was found to be negative. QuantiFERON tuberculosis testing was reactive; her tuberculin skin test was 15 mm, which was considered normal in our country. Computed tomographic scans of the neck and chest did not exhibit any abnormality. To exclude sarcoidosis, her serum angiotensin-converting enzyme level was measured and was detected as normal. Her fundus examination was also normal. Her medical history and clinical findings for berylliosis, coccidioidomycosis, and histoplasmosis were all negative.

Nine months following the operation, the patient's serum calcium level remained within the normal range (Table 1). She is still asymptomatic and has no complaints in terms of any disease. The etiology of the non-caseating granulomas has not been determined yet. The patient is keen on attending regular visits, so we choose to follow her only with watchful waiting rather than perform additional tests.

## Discussion

Granuloma-forming major cells (macrophages) produce extrarenal 1α(OH)ase, which may be the source of inappropriately high 1,25(OH)_2_D_3 _levels. Increased levels of 1α(OH)ase can cause excessive conversion of 25(OH)D_3 _to 1,25(OH)_2_D_3_. High levels of 1,25(OH)_2_D_3 _increases the absorption of calcium from the gut and resorption of calcium from bone. This inevitably results in hypercalcemia, decreased secretion of parathormone by the parathyroid glands, increased filtering load of calcium through the kidneys, and hypercalciuria [2]. The treatment consists of reducing the amount of dietary calcium, administering low-dose glucocorticoids, and avoiding sunlight exposure [2].

Our patient was found to harbor two disorders simultaneously. Her serum and urinary calcium levels were high, which could have been considered to be consistent with granuloma-associated hypercalcemia if she had had a known or suspected granulomatous disease. Her parathormone levels would be expected to be normal or suppressed in granuloma-associated hypercalcemia; however, this was not the case before surgery [2]. The decrease in her serum 1,25(OH)_2_D_3 _level was noted after removal of the adenoma. Because the serum parathormone level decreased after surgery, related hydroxylation of 25(OH)D_3 _decreased as well, resulting in reduced serum 1,25(OH)_2_D_3 _levels in our patient. But this is also inconsistent with granulomatous disease-related hypercalcemia.

Hypercalcemia due to co-existing parathyroid adenoma and sarcoidosis had been published before in the literature [3-6]; however, few cases of granulomatous inflammation within the parathyroid adenoma had been reported [7-9]. In these latter cases, the parathyroid glands had been demonstrated to be infiltrated by granulomatous inflammation of tuberculosis within parathyroid adenomas. In the report from India and in a recent report from England, in addition to granulomatous inflammation of the parathyroid adenoma, inflammation of the lymph nodes had been detected [7,9]. Our patient showed no pathology in the lymph nodes on either pathological examination or imaging, although her two excised lymph nodes showed findings consistent with reactive hyperplasia. Kar and colleagues [7] confirmed tuberculosis with a positive PCR for *Mycobacterium tuberculosis*, and Sadideen and colleagues [9] showed tuberculosis by the presence of occasional acid-fast bacilli in such cases. As it happened in our case, Jacob *et al*. [8] could not detect the origin of granulomatous inflammation, and owing to the fact that tuberculosis was common in their country, they preferred to begin anti-tuberculous treatment for their patient. Our patient was asymptomatic, so we decided not to administer empirical tuberculosis treatment, even though tuberculosis was also endemic in our country.

## Conclusions

Granulomatous infiltration of parathyroid adenoma is a rare condition. It is not clear whether the granulomatous disease contributed to the hypercalcemia of the present patient. Hypercalcemia in patients who have granulomatous disease can be managed with dietary calcium restriction, avoidance of sunlight, and low-dose glucocorticoid therapy. Even so, clinicians should keep in mind that persistent hypercalcemia might be a sign of concomitant parathyroid adenoma.

## Abbreviations

1,25(OH)2D3: Vitamin D; PCR: polymerase chain reaction; PHPT: primary hyperparathyroidism.

## Competing interests

The authors declare that they have no competing interests.

## Consent

Written informed consent was obtained from the patient for publication of this case report and any accompanying images. A copy of the written consent is available for review by the Editor-in-Chief of this journal.

## Authors' contributions

IA drafted the manuscript and gave approval of the final version to be published. ÇŞ and AL substantially contributed to the acquisition and interpretation of data. EA critically revised the manuscript. Each author participated sufficiently in the work to take public responsibility for appropriate portions of the content. All authors read and approved the final manuscript.
